# Supplementary material for: Light-Powered Reactivation of Flagella and Contraction of Microtubule Networks: Toward Building an Artificial Cell
Source: ACS Synth Biol. 2021 Mar 24;10(6):1490–504. doi: 10.1021/acssynbio.1c00071 (PMC8218302; doi:10.1021/acssynbio.1c00071)
Supplement: Supplementary file 2 — sb1c00071_si_002.pdf [file sb1c00071_si_002.pdf]

Supplementary Information for: Light-Powered Reactivation of Flagella and Contraction of Microtubule Networks: Towards Building an Artificial Cell

Raheel Ahmad,[†,#] Christin Kleineberg,[‡,#] Vahid Nasirimarekani,[¶] Yu-Jung Su,[†] Samira Goli Pozveh,[†] Albert Bae,[§] Kai Sundmacher,[‡,‖] Eberhard Bodenschatz,[†,⊥] Isabella Guido,[†] Tanja Vidaković-Koch,[*,‡] and Azam Gholami[*,†]

†*Max-Planck Institute for Dynamics and Self-Organization, Am Faßberg 17, 37077 Göttingen, Germany*

‡*Max-Planck Institute for Dynamics of Complex Technical Systems, Sandtorstraße 1, 39106 Magdeburg, Germany*

¶*Microfluidics & BIOMICS Cluster UPV/EHU, University of the Basque Country UPV/EHU, 01006, Vitoria-Gasteiz, Spain*

§*Department of Biomedical Engineering, University of Rochester, New York, USA*

‖*Otto von Guericke University, Universitaetsplatz 2, 39106 Magdeburg, Germany*

⊥*Institute for Dynamics of Complex Systems, Georg-August-University Göttingen, 37073 Göttingen, Germany*

#*These authors contributed equally.*

E-mail: vidakovic@mpi-magdeburg.mpg.de; azam.gholami@ds.mpg.de

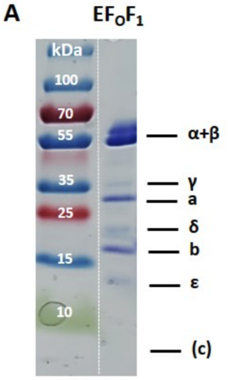

Subunit	Molecular mass (kDa)
α	55.3
β	50.3
γ	31.6
δ	19.3
ε	14.9
a	30.3
b	17.2
c	8.3

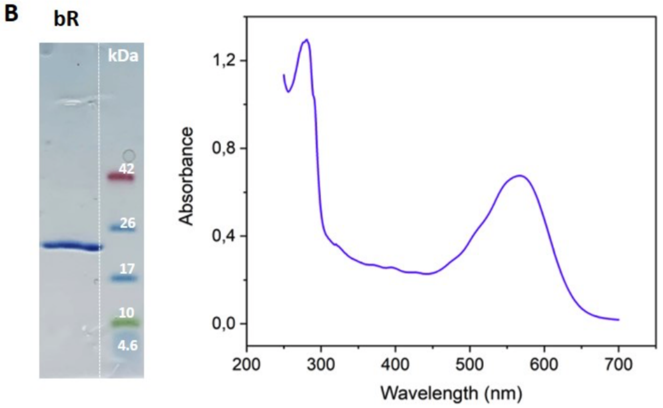

Figure S1: SDS-PAGE of purified proteins. (A) Coomassie-stained SDS-PAGE of purified EF$_O$F$_1$-ATP synthase and molecular masses of EF$_O$F$_1$ subunits. (B) Coomassie-stained SDS-PAGE of purified bR with the corresponding absorbance spectrum.

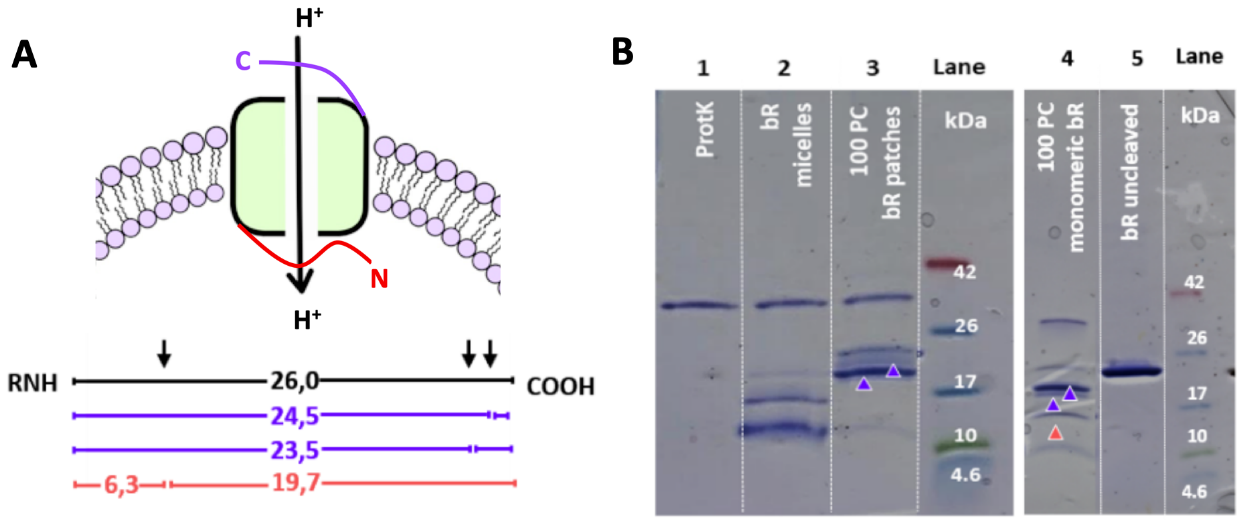

Figure S2: Proteolytic cleavage of reconstituted bR with proteinase K (ProtK) shows mixed orientation for monomeric bR and almost uniform orientation (with the c terminus outwards) for bR patches. (A) Expected sizes of proteolytic fragments for ProtK digestion of bR when the N-terminal (orange values) or C-terminal (violet values) is exposed to the bulk solution (B) SDS-PAGE gel analysis of the digestion products. Lane 1: band specific for ProtK enzyme only; lane 2: digestion product of not reconstituted bR; lane 3: digest pattern for PC vesicles containing bR patches; lane 4: digest pattern for PC vesicles containing monomeric bR; lane 5: undigested bR in PC lipid vesicles. Part of this data was previously published in Ref.[1].

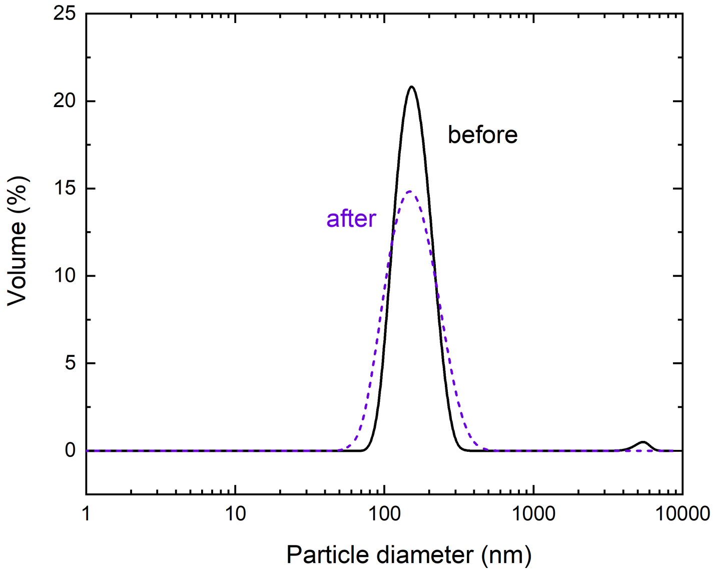

Figure S3: Size distribution of vesicles before (before) and after reconstitution and removal of detergent using Bio Beads (after).

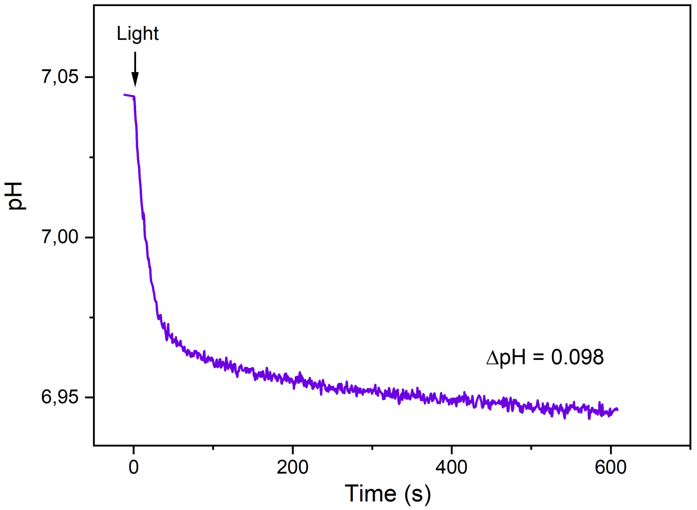

Figure S4: Proton pumping of bR as measured by encapsulated pyranine.

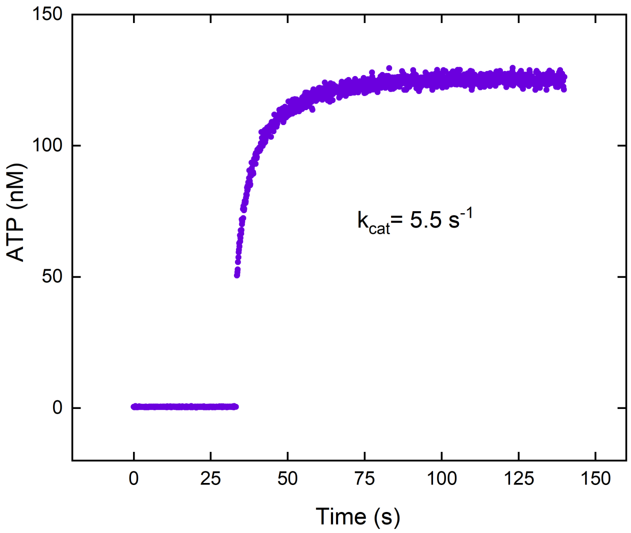

Figure S5: Turnover (k_{cat}) of ATP synthase as determined in an acid-base transition experiment. Measurements were performed in 20 mM succinate-NaOH, 5 mM NaH_2PO_4, 0.6 mM KOH (inner solution) and 200 mM tricine-NaOH, 5 mM NaH_2PO_4, 160 mM KOH (outer solution) adjusted with 18 µM valinomycin at room temperature. [ADP]= 0.1 µM, [P^i]= 5 mM, [lipid]= 0.24 mg/mL, [EF_0F_1]= 2.22 nM, $\Delta\Psi$= 143 mV. ATP synthase was reconstituted with 0.8 % Triton.

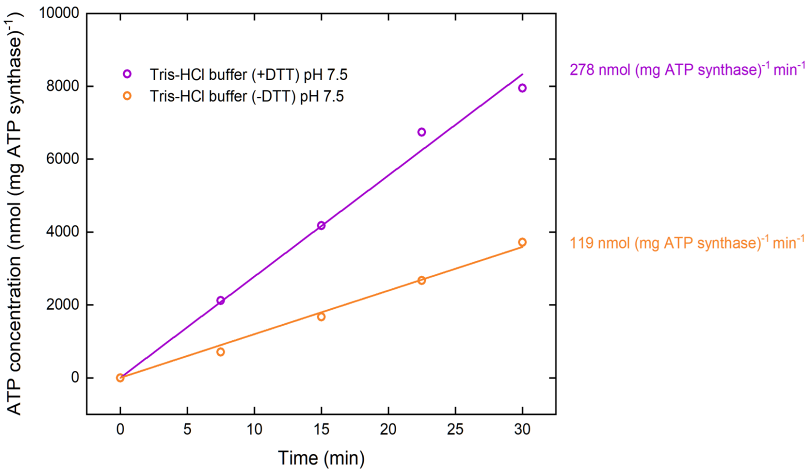

Figure S6: ATP production in Tris-HCl buffer in the presence (+DTT) and absence of DTT (-DTT). DTT conserves proteins in their functional form as it prevents oxidation of sulfhydryl groups (SH-) to disulfide-bonds in the presence of air oxygen. The activity was determined by linear regression. Measurements were performed with [ADP]= 300 µM, [P_i]= 5 mM, [lipid]= 0.022 mg/mL, [EF_0F_1]= 1.3 nM, [bR]= 88 nM, $\Delta\Psi$= 143 mV at room temperature. Proteins were reconstituted with 0.8 % Triton.

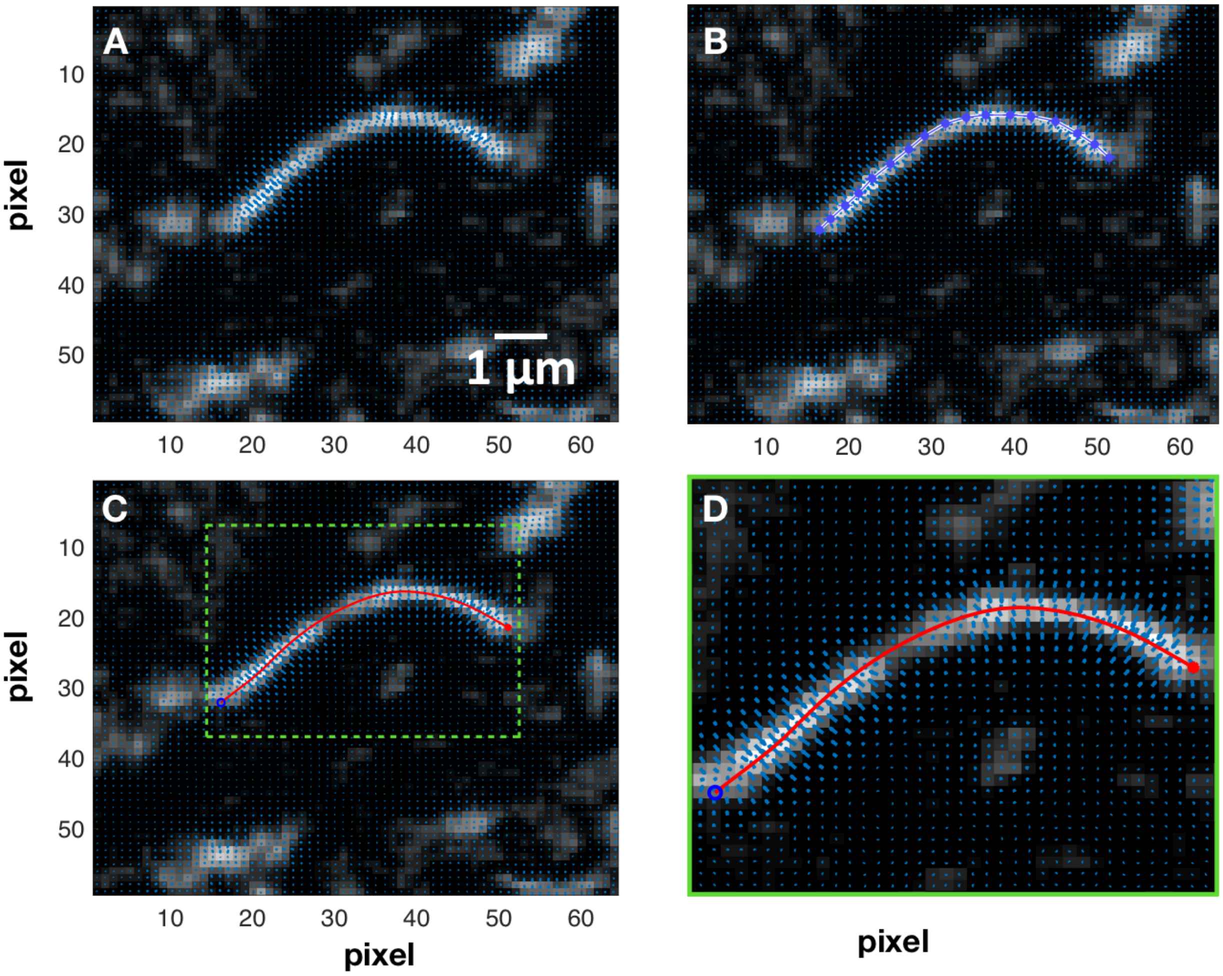

Figure S7: A) Gradient vector flow calculated at the vicinity of an axoneme. B) The initial selection of a polygon for the first frame which deforms according to the gradient vector flow. C) The final tracked shape of an axoneme. D) A zoomed-in image showing the gradient vector flow (blue arrows) at a higher magnification.

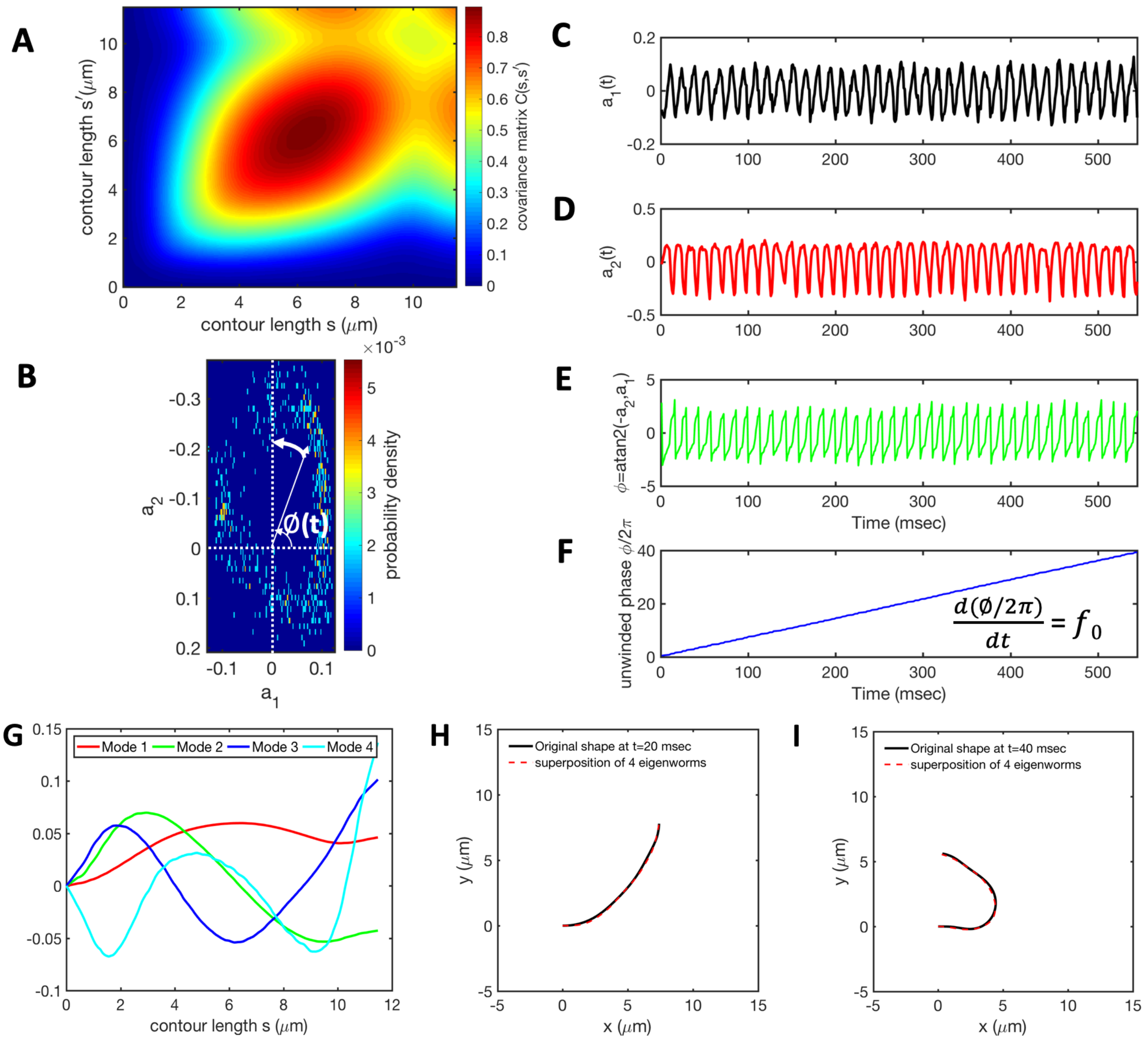

Figure S8: Mode analysis of the reactivated axoneme presented in Figure 6A-E. A) The covariance matrix $C(s, s')$ of fluctuations in angle $\theta(s,t)$. B) The probability distribution of the first two shape amplitudes $p(a_1(t), a_2(t))$. The phase angle of the axoneme as an oscillator is defined as $\phi(t) = \text{atan2}(-a_2(t), a_1(t))$. C-D) Time evolution of the first two dominant shape amplitudes $a_1(t)$ and $a_2(t)$ showing regular oscillations at frequency of 72 Hz. E-F) We observe a linear growth in dynamics of $\phi(t)$, indicating steady rotation in $a_1 - a_2$ plane presented in part B. Note that $d\phi/dt = 2\pi f_0$ where f_0 is the beating frequency of axoneme. G) Four eigenvectors corresponding to the four largest eigenvalues of matrix $C(s, s')$. H-I) Superposition of four eigenmodes presented in part G with coefficients $a_1(t)$ to $a_4(t)$, can reproduce shape of the axoneme with high accuracy.

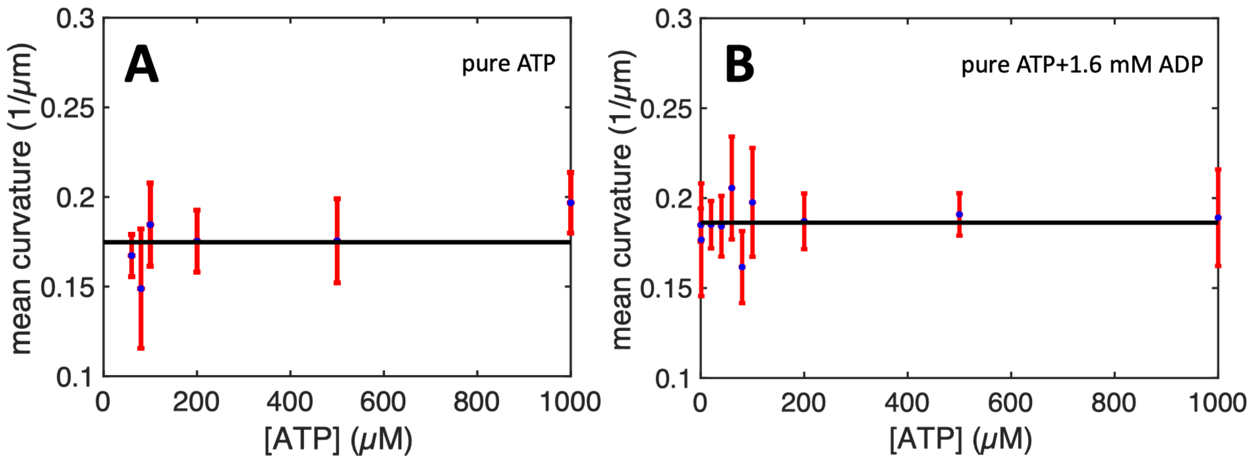

Figure S9: Mean curvature of axonemes reactivated using pure commercial ATP plotted versus ATP concentration. In panel A, no ADP is added ,while in panel B, the reactivation buffer is supplemented with 1.6 mM ADP. The black solid lines show the mean values of 0.17 μm^{-1} (A) and 0.186 μm^{-1} (B), which is comparable to the value of 0.16 μm^{-1} in energy module experiments shown in Fig. 4E. The corresponding frequency trends in pure ATP experiments with and without ADP are shown in Fig. 5C and Fig. 3E, respectively.

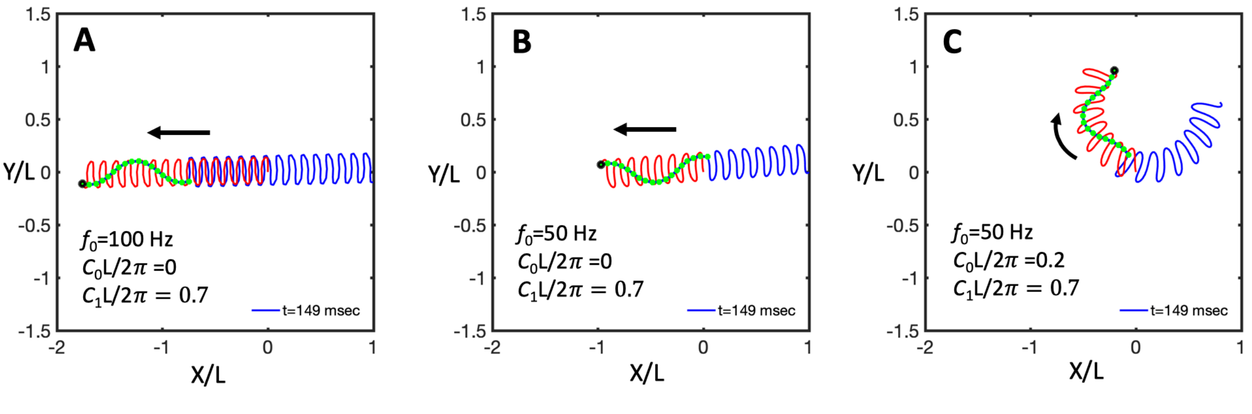

Figure S10: A-B) A flagellum swimming in the absence of static curvature C_0 at two different frequencies of 100 and 50 Hz. Faster beating flagellum swims a longer distance. C) Flagellum follows a circular path if C_0 is non-zero. Amplitude of the dynamic mode C_1 is kept the same for all three simulations. L is the contour length of flagellum (see Video 8).

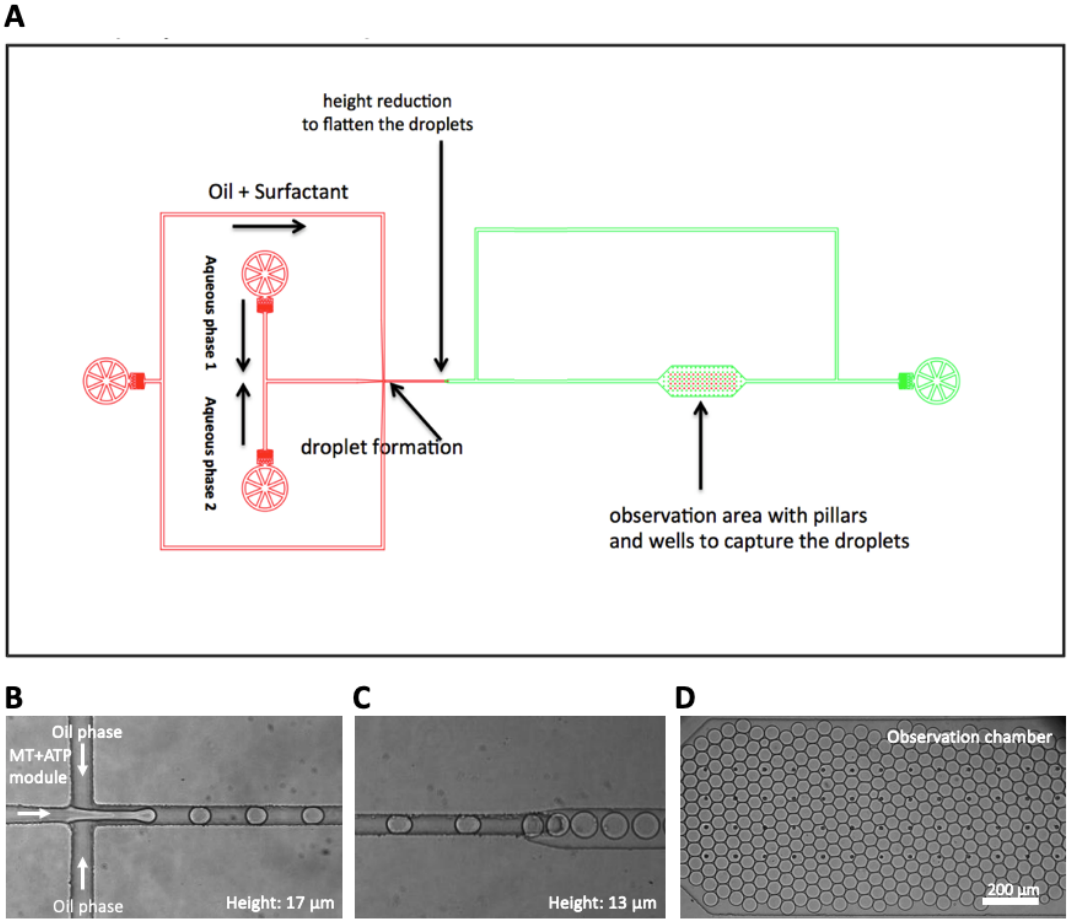

Figure S11: Droplet microfluidic setup. A) Schematic diagram showing the encapsulation of MTs/kinesin-1 network (aqueous phase 1) with light-driven ATP module (aqueous phase 2). B) Mono-disperse water-in-oil droplets containing MTs/kinesin-1 solution and light-driven ATP module are formed at the T-junction. C) Droplets enter the green area where height is decreased from 17 µm to 13 µm. D) Observation chamber with entrapped droplets where the flow into the chamber was stopped by guiding the flow to the side channel. The following flow rates were adjusted: aqueous phase 1, 200 μL/h; aqueous phase 2, 200 μL/h; oil phase, 400 μL/h.

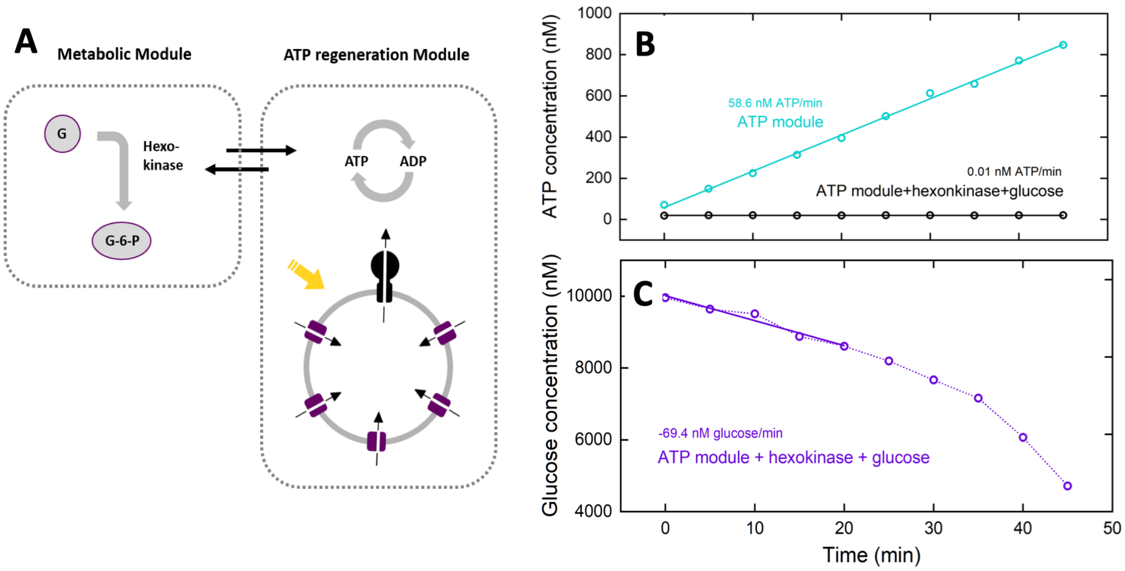

Figure S12: ATP and glucose concentration over time under green light illumination. A) Scheme of coupling the ATP regeneration module with a metabolic module for glucose consumption triggered by light. B-C) Measurements were performed in 20 mM tricine-NaOH, 20 mM succinate, 0.6 mM KCl, 80 mM NaCl (inner solution) and 200 mM tricine-NaOH, 5 mM NaH_2PO_4, 160 mM KOH, pH 8.8 (outer solution) in the presence of 20 µM valinomycin at room temperature. [ADP]= 180 µM, [P_i]= 5 mM, [lipid]= 0.022 mg/mL, [EF_OF_1]= 1.3 nM, [bR]= 88 nM, $\Delta\Psi$= 143 mV. Proteins were reconstituted with 0.8 % Triton. For measurement of glucose consumption 10 µM glucose and 2.7 µg/mL hexokinase (2400 U/mL) was added to the outer solution. Glucose concentration was determined using a highly sensitive glucose assay (Sigma).

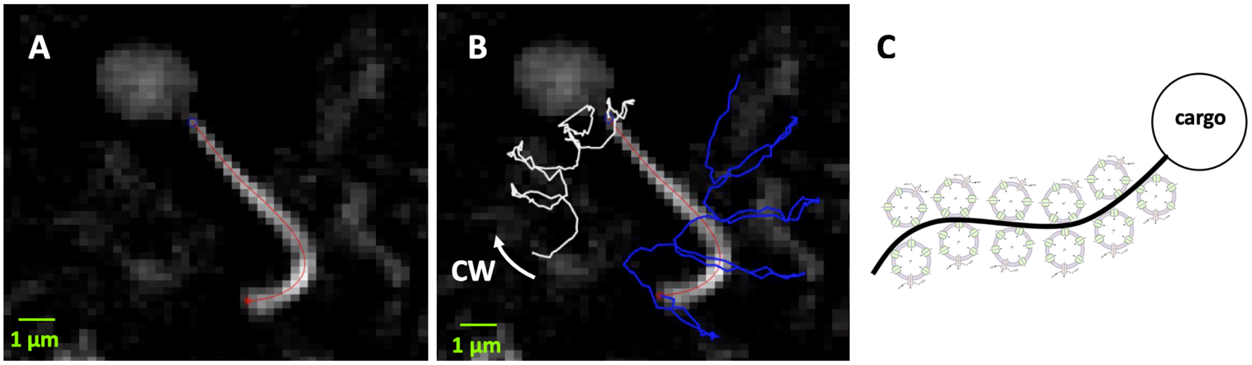

Figure S13: Bead as a cargo. A) A bead of radius 1 μm is attached to the distal end of an axoneme. B) Upon illuminations, the reactivated axoneme propels the bead. Blue line shows the trace of the basal end and the white line is the trace of the distal tip which is attached to a bead (SI, Video 11). C) A cartoon showing that an enhanced vesicle-axoneme attachment, e.g. via electrostatic interactions, could be beneficial for the local production of ATP in the vicinity of axonemes.

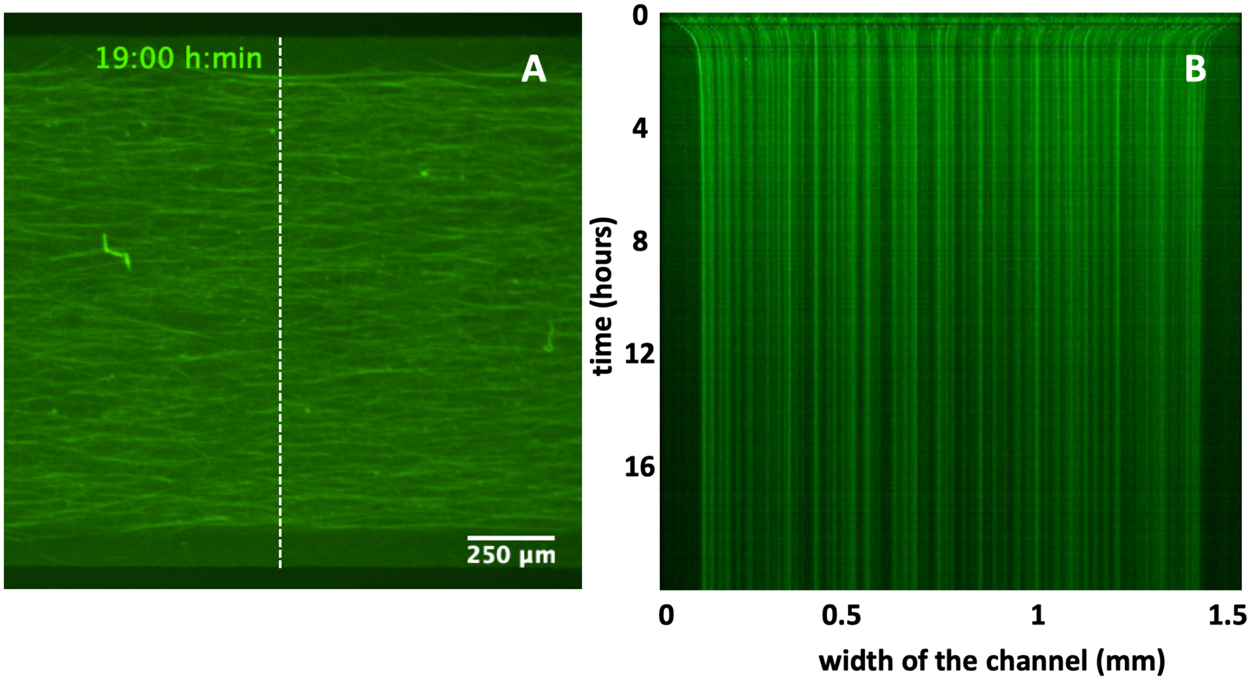

Figure S14: A control experiment with 1 mM pure commercial ATP to confirm that a contracted MTs/kinesin1 network does not relax back to its initial state once the ATP is depleted. A) Snapshot of the millifluidic set up filled with MTs/kinesin1 network after 19 hours. B) The space-time plot along the white dashed line in panel B showing the network contraction. Note that the cross-linked network remains contracted for a long period of time (see Video 12).

List of supplementary videos

Video 1 Reactivation of axonemes with 80 μM pure commercial ATP.

Video 2 Swimming trajectory of an axoneme selected from Video 1.

Video 3 Axonemes reactivated with energy module under illumination.

Video 4 Swimming trajectory of an axoneme selected from Video 3.

Video 5 Comparison of beating frequencies of axonemes reactivated with energy module which is pre-illuminated for different time periods.

Video 6 Attachment of functionalized vesicles to the part of the contour length of an actively beating axoneme.

Video 7 Swimming trajectory of an axoneme in the presence of 1 mM $CaCl_2$.

Video 8 Simulations of the swimming trajectories of an axoneme for different parameters.

Video 9 Contraction of MTs/kinesin1 network mixed with the energy module and encapsulated inside droplets.

Video 10 Contraction of MTs/kinesin1 network mixed with the energy module inside a millifluidic device.

Video 11 Swimming trajectory of an axoneme attached to a bead of diameter 1 μm as a cargo.

Video 12 Contraction of MTs/kinesin1 network mixed with 1mM pure ATP inside a millifluidic device.

Video 13 MTs/kinesin1 network mixed with the non pre-illuminated energy module fails to contract.

References

(1) Kleineberg, C.; Wölfer, C.; Abbasnia, A.; Pischel, D.; Bednarz, C.; Ivanov, I.; Heitkamp, T.; Börsch, M.; Sundmacher, K.; Vidakovic-Koch, T. Light-driven ATP regeneration in diblock/grafted hybrid vesicles. *ChemBioChem* **2020**, *21*, 2149–2160.
